# Clinical value of SLC12A9 for diagnosis and prognosis in colorectal cancer

**DOI:** 10.18632/aging.205360

**Published:** 2023-12-28

**Authors:** Wang Du, Guozhi Xia, Liang Chen, Lingjun Geng, Rubin Xu, Qingqing Han, Xiaomei Ying, Hongzhu Yu

**Affiliations:** 1 Department of General Surgery, Fuyang Hospital of Anhui Medical University, Fuyang, Anhui 236000, China; 2 Department of Hepatobiliary and Pancreatic Surgery, Conversion therapy center for Hepatobiliary and Pancreatic Tumors, First Hospital of Jiaxing, Affiliated Hospital of Jiaxing University, Jiaxing, Zhejiang 314041, China; 3 Suzhou Hospital of Anhui Medical University, Suzhou, Anhui 234000, China

**Keywords:** colorectal cancer, *SLC12A9*, single cell, diagnosis, prognosis

## Abstract

Objective: The goal of the study is to assess the clinical value and the potential mechanism of *SLC12A9* combing transcriptome and single cell sequencing data.

Methods: In this study, the expression level and the receiver operating characteristic curve analysis of *SLC12A9* in CRC and normal tissue were analyzed in multiple data cohort. The standardized mean difference (SMD) calculation and the summary receiver operating characteristic (SROC) analysis were performed further to detect its diagnostic ability and expression level. KM survival analysis was performed to assess the prognosis value of *SLC12A9*. The expression level of *SLC12A9* in different clinical characteristics was analyzed to explore the clinical value. Single cell data was studied to reveal the potential mechanism of *SLC12A9*. The correlation analysis of immunoinfiltration was performed to detect the potential immune cell related to *SLC12A9*. The nomogram was drawn to assess the probable mortality rate of CRC patient.

Results: We found that *SLC12A9* was significantly up-regulated with the moderate diagnostic value in CRC. Patients with overexpressed *SLC12A9* had a worse prognosis. *SLC12A9* was related to Age, Pathologic N stage, Pathologic M stage, Lymphatic invasion and Pathologic stage (*p* < 0.05). The 1, 3 and 5-year survival rates of patient named TCGA-G4-6309 are 0.959, 0.897 and 0.827. PCR also showed that *SLC12A9* was overexpressed in CRC comparing with normal tissue.

Conclusion: In conclusion, our study comprehensively analyzed the clinical value of SLC12A9 and its potential mechanism, as well as immune cell infiltration, which may accelerate the diagnosis and improve the prognosis of CRC.

## INTRODUCTION

A significant global health challenge is presented by colorectal cancer (CRC), with high mortality rates and an urgent requirement for improved diagnostic and therapeutic strategies [1, 2]. Successful treatment outcomes rely heavily on early detection, but the lack of reliable biomarkers currently poses a significant obstacle [3]. Thankfully, recent progress in transcriptome analysis provides a promising avenue for the identification of potential biomarkers and the unraveling of the complex molecular mechanisms involved in CRC development [4]. Metabolic reprogramming is a key characteristic of CRC, involving changes in the energy metabolism of cancer cells [5]. This reprogramming has generated increased interest in the role of solute carrier (SLC) transporters, a protein family accountable for the transportation of various metabolites across cell membranes [6].

Cancer, including colorectal cancer, is characterized by metabolic reprogramming [7]. Altered metabolic phenotypes are observed in cancer cells to fulfill their heightened energy requirements for rapid growth and survival [8]. In colorectal cancer (CRC), a significant metabolic change known as the Warburg effect occurs [9]. This effect involves an increased dependence on glycolysis, even in the presence of oxygen, leading to higher lactate production [10]. This metabolic shift not only supplies cancer cells with ample energy but also aids in the generation of metabolic intermediates essential for biosynthesis and maintaining redox homeostasis [11].

Alongside increased glycolysis, dysregulated nutrient uptake and utilization are observed in CRC cells, regulated by SLC transporters [12]. These transporters play vital roles in the cellular acquisition of nutrients, including glucose, amino acids, vitamins, and ions [13]. In CRC, specific SLC transporters exhibit disrupted expression and activity, indicating their potential as targets for diagnosis and treatment [14]. For instance, CRC is associated with elevated expression of glucose transporters (GLUTs), particularly GLUT1 and GLUT3, which enhance glucose uptake and metabolism [15]. Similarly, altered expression of amino acid transporters like *SLC1A5* and *SLC7A5* is linked to increased uptake of amino acids, fueling the anabolic processes required for tumor growth and survival [16, 17].

The dysregulation of SLC transporters in CRC offers a promising prospect for the discovery of new biomarkers [18]. RNA sequencing and other transcriptome analysis methods allow researchers to study the gene expression patterns of cancer cells and compare them to those of healthy tissue samples [19]. By employing these analyses, changes in the expression of SLC transporters and related metabolic genes can be detected, leading to the identification of potential biomarker candidates.

The potential of SLC transporters as CRC biomarkers has already been established through various studies. For example, the upregulation of *SLC2A1* (encoding GLUT1) has been linked to advanced stages of CRC, unfavorable prognosis, and enhanced resistance to chemotherapy [20]. Additionally, overexpression of *SLC6A14*, an amino acid transporter, has been associated with lymph node metastasis and unfavorable survival outcomes in CRC patients [21]. These findings indicate that the expression levels of certain SLC transporters can serve as indicators of disease progression and patient prognosis.

Furthermore, the dysregulation of SLC transporters in CRC holds potential for targeted therapies [22]. By leveraging the abnormal expression of these transporters, it may be feasible to devise innovative therapeutic approaches that specifically disrupt the metabolic weaknesses of cancer cells while preserving normal tissues [23]. For instance, preclinical studies have demonstrated promising outcomes by targeting GLUT transporters in conjunction with conventional chemotherapy or radiotherapy. This combination approach has enhanced treatment effectiveness and surmounted drug resistance.

This study aimed to explore the involvement of *SLC12A9* in colorectal cancer (CRC) through multiple analysis techniques, including expression analysis and prognostic evaluation. The objective was to investigate the expression patterns of *SLC12A9* and determine its potential as a prognostic indicator and therapeutic target in CRC. The findings aimed to provide insights into the significance of *SLC12A19* as a potential biomarker and therapeutic target in CRC.

## METHODS

### Data download and processing

The transcriptome dataset used in this study to investigate colorectal cancer (CRC) was obtained from the TCGA and GEO databases. The dataset was selected based on specific inclusion criteria, including: (1) the tissue samples were of human origin, (2) there were more than 6 tumor and normal tissue samples, (3) the expression data included *SLC12A9*, and (4) the platform annotation file allowed for the conversion of probe names to gene names. The dataset underwent filtration based on these conditions, and all data were log-standardized for subsequent analysis. Additionally, for single-cell analysis of colorectal cancer, the dataset GSE188711 from the GEO database was utilized [24]. This dataset contains the transcriptomes of 27,927 cells from 3 left and 3 right CRC patients. In this study, the authors found differences in T-cell exhaustion between left-sided and right-sided colon cancer and differences in immunotherapy.

### Quality control and processing of single cell sequencing data

In this study, the single-cell sequencing data were processed and analyzed using the “Seurat” R package. Specific screening criteria were applied to both genes and cells, which included the following steps: (1) Genes expressed in less than 3 cells were removed. (2) Cells with less than 200 gene expressions were excluded. (3) Cells expressing more than 6000 genes were excluded. (4) Cells with a mitochondrial gene percentage higher than 0.15 were removed. (5) Cells with a total gene expression value exceeding 100,000 were removed.

For data integration, the “SCTransform” method was employed, with a high-variable gene threshold set at 3000. Standardization was performed using the “SCT” method. The dimensions were reduced using the RunTSNE function, with the “dims” parameter set to 20. Clustering of the samples was conducted with the parameters “k.perl = 20, resolution = 0.6, algorithm = 3, random.seed = 2023”.

Cell types were annotated based on marker genes described in published literature, and the results were visualized using a t-distributed stochastic neighbor embedding (TSNE) map.

### The expression of SLC12A9 was investigated in colon cancer and normal tissues

The R software version 4.2.2 was utilized in this study. The expression of *SLC12A9* in normal and colorectal cancer samples in each dataset was examined using the independent sample *t*-test. For visualization of the results, the “ggplot2” package was employed, presenting the findings through boxplots.

### Clinical diagnostic value of SLC12A9 gene

ROC analysis of colorectal cancer and normal samples from each dataset was conducted using the “pROC” package. The results of the analysis were visualized to provide a graphical representation of the performance of the classifier.

### The meta-analysis of different cohorts

For the meta-analysis, STATA 14.0 software and the “meta” R package were utilized. The standard mean deviation (SMD) was calculated to assess the expression level of *SLC12A9* in colorectal cancer. In cases where significant heterogeneity was present (I^2^ > 0.50) among the included datasets, the random-effects model was selected for the analysis. To detect publication bias, the funnel function of the “meta” R package was employed, with a *p*-value ≥ 0.05 indicating no publication bias. Furthermore, the diagnostic value of *SLC12A9* was assessed using the summary receiver operating characteristic (sROC) curve. Specificity and sensitivity measures were calculated to evaluate the diagnostic performance of *SLC12A9*.

### Prognostic and clinical correlation analysis of SLC12A9

Based on the median value of *SLC12A9* gene expression, the samples were categorized into high and low expression groups. The Kaplan-Meier (KM) survival analysis method was employed to generate survival curves based on these groups. Furthermore, the expression of *SLC12A9* was examined in relation to different clinical characteristics, including gender, age, T stage, N stage, M stage, lymph node metastasis, and pathological stage. The rank-sum test was utilized to assess the significance of the differences in *SLC12A9* expression among these clinical characteristics.

### Gene set enrichment analysis

To identify the signaling pathways associated with *SLC12A9* in colorectal cancer, Gene Set Enrichment Analysis (GSEA) was conducted. Initially, the differences between the high and low *SLC12A9* expression groups were analyzed. The genes were then sorted in descending order of log2 fold change (log2FC) using the clusterProfiler package. Pathways with an adjusted *p*-value below 0.05 were considered enriched, indicating their potential association with *SLC12A9* in colorectal cancer.

### Correlation analysis of immune cell infiltration

In this study, the CIBERSORT method was employed to calculate the immune infiltration in each colorectal cancer sample. The rank sum test was then utilized to investigate the immune cell infiltration between the Low_*SLC12A9* and High_*SLC12A9* groups. Furthermore, the correlation between immune cells and *SLC12A9* was analyzed using the “spearman” method. This analysis aimed to examine the relationship between the expression of *SLC12A9* and the presence of immune cells in the tumor microenvironment of colorectal cancer.

### Pseudo-time series analysis

In this study, the pseudo-time series analysis was conducted using the monocle2 package. Specifically, all immune cells were extracted for analysis. The parameters “max_components” were set to 2, and the method used was “DDRTree.” The results of the analysis were visualized using the plot_cell_trajectory function, which provided insights into the developmental trajectories of immune cells in colorectal cancer.

### The construction of a nomogram

In this study, the “regplot” package was employed to integrate the expression of the gene *SLC12A9* with other clinical features. This integration facilitated the construction of a nomogram, which was utilized to predict the 1, 3, and 5-year survival outcomes in colorectal cancer. The nomogram provided a graphical representation of the predictive model, incorporating multiple factors including *SLC12A9* expression and clinical features to estimate survival probabilities at different time points.

### The expression of SLC2A9 was verified by PCR

Next, we performed qRT-PCR assay on 6 pairs of colorectal cancer samples to obtain mRNA quantization from colorectal cancer and adjacent tissues. All six patients signed informed consent forms. This study was approved by the Ethics Committee of Fuyang Hospital affiliated to Anhui Medical University. Total cellular RNA was isolated from cells using Trizol Reagent (Invitrogen, Carlsbad, CA, USA) according to manufacturer’s instructions. Reverse transcription was performed using a reverse transcription kit provided by Takara (Otsu, Shiga, Japan). Real-time polymerase chain reaction (RT-PCR) was performed using a quantitative SYBR Green PCR kit (Takara) and Applied Biosystems QuantStudio 1 (Thermo Fisher, Waltham, MA, USA). The relative quantitative determination was performed by −2ΔΔCt method. The relative mRNA expression of each gene was normalized to the level of glyceraldehyde-3-phosphate dehydrogenase (GAPDH) mRNA. PCR uses the following primer sequence:

**Table:** 

*SLC12A9*:	
Forward	TGGCTATGCTGAGGACTA
Reverse	ATAGACGAAGAAGGTGTAGG
*GAPDH*:	
Forward	GAACGGGAAGCTCACTGG
Reverse	GCCTGCTTCACCACCTTCT

### Statistics analysis

Bioinformatics analysis was performed in R software (version 4.2.2). The Seurat package was used for single-cell sequencing analysis. The expression of SLC12A9 was analyzed using an independent sample *t*-test. *p* < 0.05 was considered statistically significant.

## RESULTS

### Sample information for the datasets

A total of 10 data sets have been gathered, which encompass databases such as TCGA and GEO. The particulars of these data sets are provided in Table 1. Each dataset comprises data pertaining to both colorectal cancer and normal control. In order to analyze *SLC12A9*, the mean (Mean) and standard deviation (SD) were computed for both colorectal cancer and normal tissue samples.

**Table 1:** Characteristics of TCGA and GEO datasets included in the study.

**GSE number**	**Platform**	**NO**	**MO**	**SDO**	**N1**	**M1**	**SD1**	**Total**
TCGA Cohort	NR	51	2.54	0.323	616	3.26	0.538	667
GSE8671	GPL570	32	7.44	0.281	32	7.86	0.305	64
GSE10972	GPL6104	24	10.4	0.337	24	11	0.552	48
GSE41657	GPL6480	12	−0.321	0.284	25	0.315	0.4	37
GSE73360	GPL17586	31	6.36	0.212	55	6.72	0.224	86
GSE84984	GPL17586	6	6.12	0.206	9	6.79	0.177	15
GSE106582	GPL10558	117	8.64	0.3	77	9.17	0.506	194
GSE110224	GPL570	17	6.22	0.174	17	6.41	0.15	34
GSE113513	GPL15207	14	7.1	0.214	14	7.61	0.368	28
GSE156355	GPL21185	6	9.49	0.084	6	10	0.323	12

Abbreviations: NO: normal tissue sample number; M0 and SD0: mean and standard deviation of normal (non-tumor) tissues; M1 and SD1: mean and Standard deviation of colorectal cancer; Total: total sample number.

### Expression of SLC12A9 in colorectal cancer and normal tissue samples

**Figure 1:**
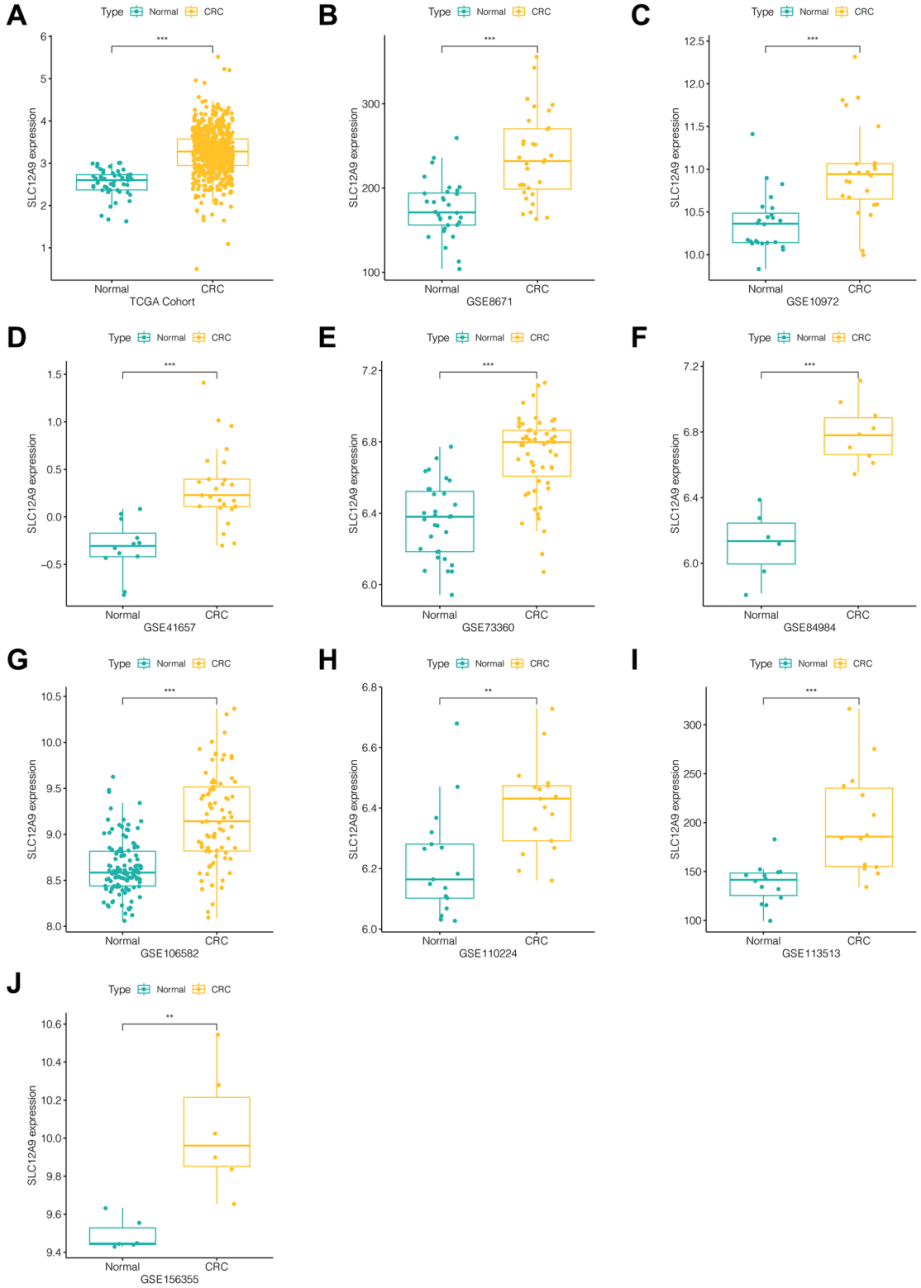
**Expression of *SLC12A9* in different cohorts.** (**A**) TCGA cohort. (**B**) GSE8671. (**C**) GSE10972. (**D**) GSE41657. (**E**) GSE73360. (**F**) GSE84984. (**G**) GSE106582. (**H**) GSE110224. (**I**) GSE113513. (**J**) GSE156355.

In the initial phase of this study, the expression of *SLC12A9* was examined in tumor and normal tissues within each data set. Figure 1A–1J illustrates the findings, indicating that *SLC12A9* exhibited an increased expression pattern in colorectal cancer across all data sets (TCGA cohort, GSE8671, GSE10972, GSE41657, GSE73360, GSE84984, GSE106582, GSE110224, GSE113513, GSE156355), in comparison to normal tissues (^*^p < 0.05, ^**^p < 0.01, ^***^p < 0.001).

### Clinical diagnostic value of SLC12A9 gene

**Figure 2:**
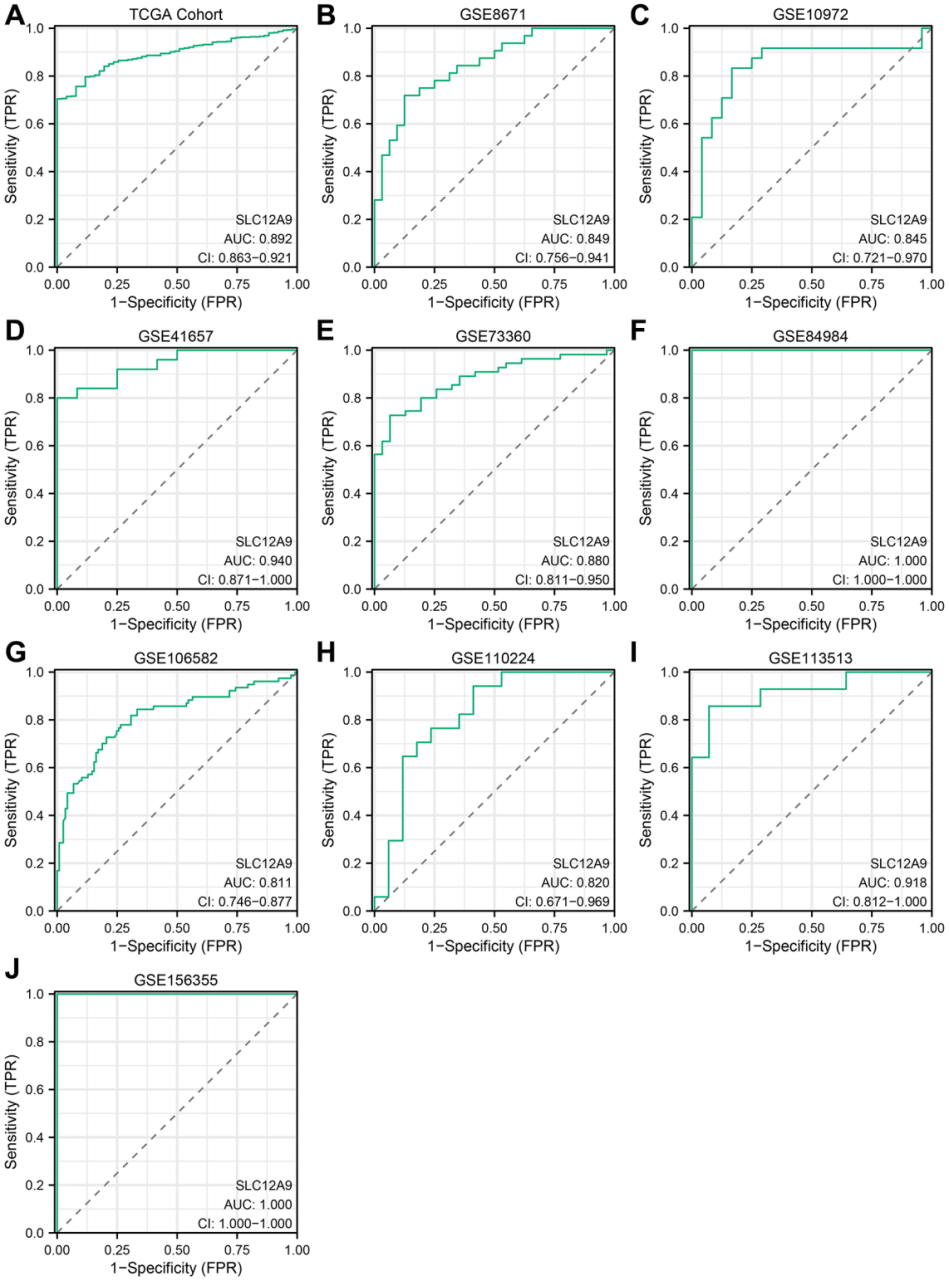
**ROC curve of *SLC12A9* in different cohorts.** (**A**) TCGA cohort. (**B**) GSE8671. (**C**) GSE10972. (**D**) GSE41657. (**E**) GSE73360. (**F**) GSE84984. (**G**) GSE106582. (**H**) GSE110224. (**I**) GSE113513. (**J**) GSE156355.

The study further investigated the diagnostic potential of *SLC12A9* in colorectal cancer. Figure 2A demonstrates that in the TCGA dataset, the area under the curve (AUC) was 0.892, indicating a moderate diagnostic value. Similarly, in Figure 2B–2J, specifically in data sets GSE8671, GSE10972, GSE73360, GSE106582, and GSE110224, the AUC values exceeded 0.8, signifying a substantial diagnostic value. Moreover, in data sets GSE41657, GSE84984, GSE113513, and GSE156355, the AUC values surpassed 0.9, indicating a high diagnostic value.

### The meta-analysis of SLC12A9 in multiple cohorts

**Figure 3:**
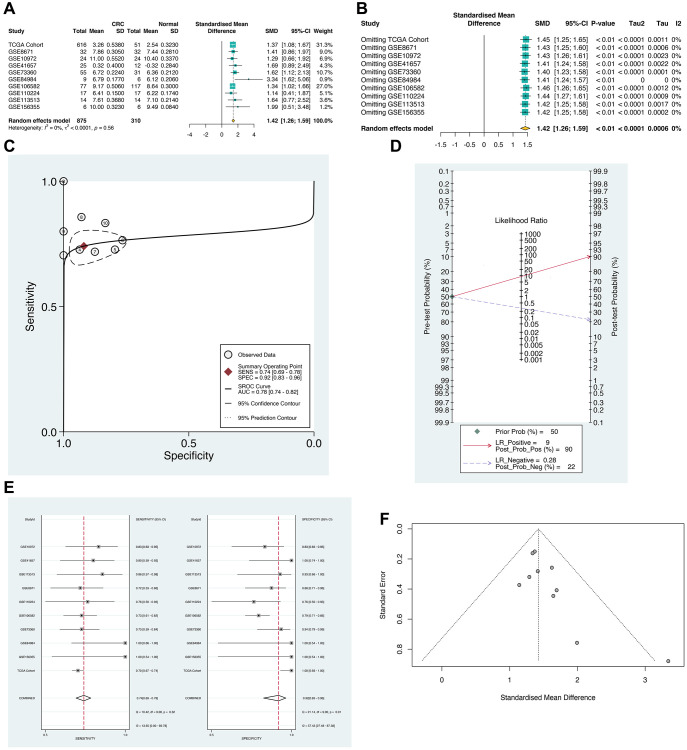
**The meta-analysis of *SLC12A9* in multiple cohorts.** (**A**) Meta analysis result. Heterogeneity test. I^2^ = 68.6%. The results showed the high expression of *SLC12A9* in colorectal cancer (SMD = 1.42, CI: 1.26-1.59). (**B**) Results of meta analysis after omitting. (**C**) sROC analysis. (**D**) Fagan’s diagram. (**E**) sROC analysis. (**F**) Detection of publication bias.

Following that, a comprehensive analysis of the expression and clinical diagnostic value of the *SLC12A9* gene was conducted. Figure 3A, 3B illustrates the results of the heterogeneity test, indicating significant heterogeneity with I^2^ = 68.6% (*p*-value < 0.05). To calculate the standardized mean difference (SMD), a random effects model was utilized, revealing a high expression of *SLC12A9* in colorectal cancer (SMD = 1.42, 95%CI: 1.26–1.59). Additionally, sROC analysis was performed, as shown in Figure 3C, 3E, which yielded an AUC of 0.78 (95%CI: 0.74–0.82), sensitivity of 0.92 (95% CI: 0.83–0.96), and specificity of 0.74 (95% CI: 0.69–0.78), indicating that *SLC12A9* possesses a moderate diagnostic value in colorectal cancer. Furthermore, Figure 3D presents Fagan’s diagram, where at a pre-detection probability of 50%, the probability of positive colorectal cancer detection using *SLC12A9* was 90%, and the probability of negative colorectal cancer detection was 28%. Figure 3F shows no significant publication bias. These findings suggest that *SLC12A9* holds potential as an effective biomarker for colorectal cancer.

### Prognostic and clinical correlation analysis of SLC12A9 gene

**Figure 4:**
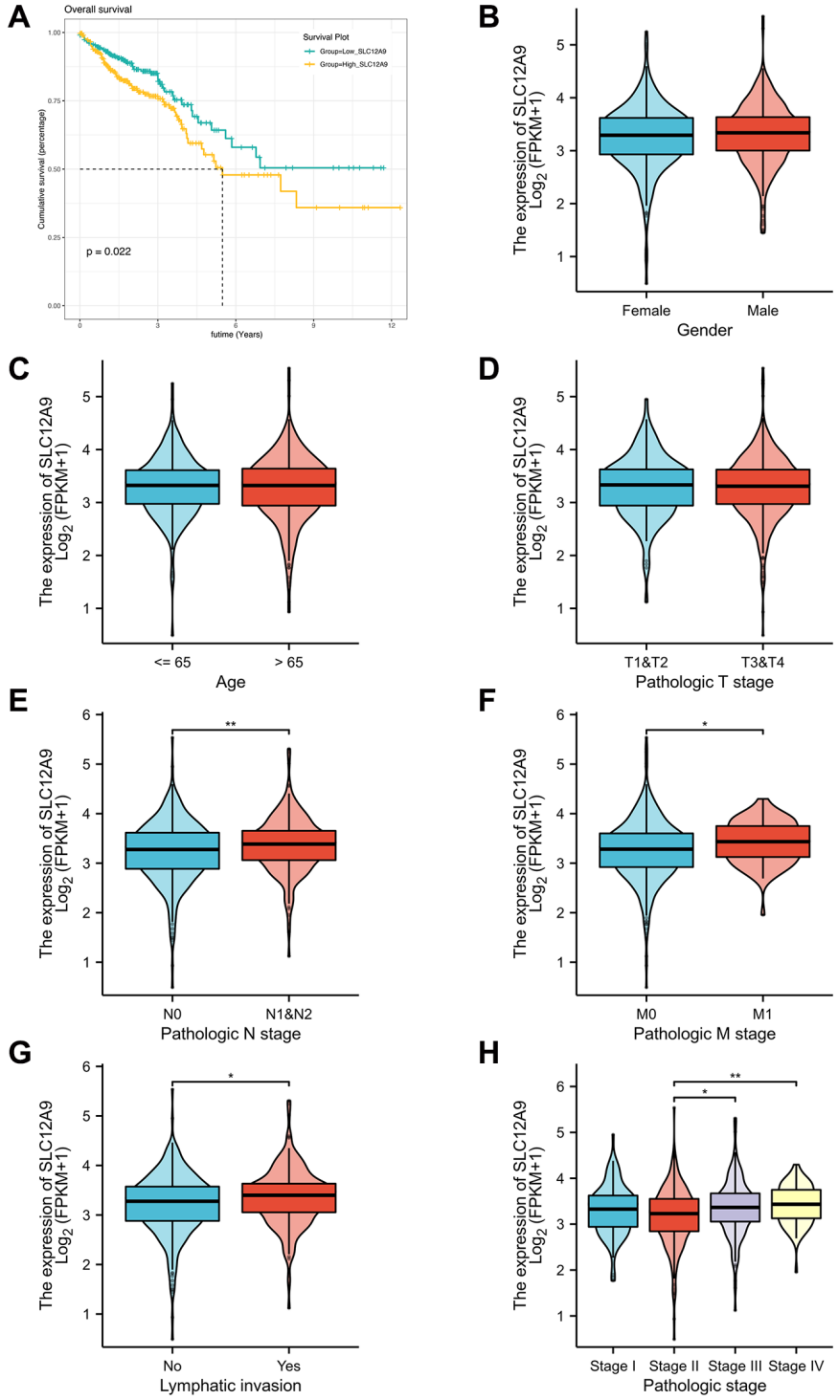
**Prognostic and clinical correlation analysis of *SLC12A9* gene.** (**A**) Survival analysis showed that high expression of *SLC12A9* was associated with poor prognosis in colorectal cancer. (**B**–**D**) There were no significant abnormalities in *SLC12A9* expression based on gender, age, and T stage of the patients. (**E–H**) *SLC12A9* expression was up-regulated in patients with N1&N2 compared to N0 patients (*p* < 0.01). Additionally, the expression of *SLC12A9* was elevated in M1 patients compared to M0 patients (*p* < 0.05). Moreover, *SLC12A9* expression was higher in patients with lymph node metastasis compared to those without, and it was also higher in Stage III and Stage IV patients compared to Stage II patients (*p* < 0.05).

The impact of *SLC12A9* on the prognosis of colon cancer has been investigated, as depicted in Figure 4A. Notably, patients in the high *SLC12A9* group exhibited a poorer prognosis (*p* < 0.05). Subsequently, the expression of the *SLC12A9* gene in patients with different clinical characteristics was examined. Figure 4B–4D demonstrates that there were no significant abnormalities in *SLC12A9* expression based on gender, age, and T stage of the patients. However, in Figure 4E–4H, it is evident that *SLC12A9* expression was up-regulated in patients with N1&N2 compared to N0 patients (*p* < 0.01). Additionally, the expression of *SLC12A9* was elevated in M1 patients compared to M0 patients (*p* < 0.05). Moreover, *SLC12A9* expression was higher in patients with lymph node metastasis compared to those without, and it was also higher in Stage III and Stage IV patients compared to Stage II patients (^*^p < 0.05, ^**^p < 0.01).

### Single cell analysis and enrichment analysis

**Figure 5:**
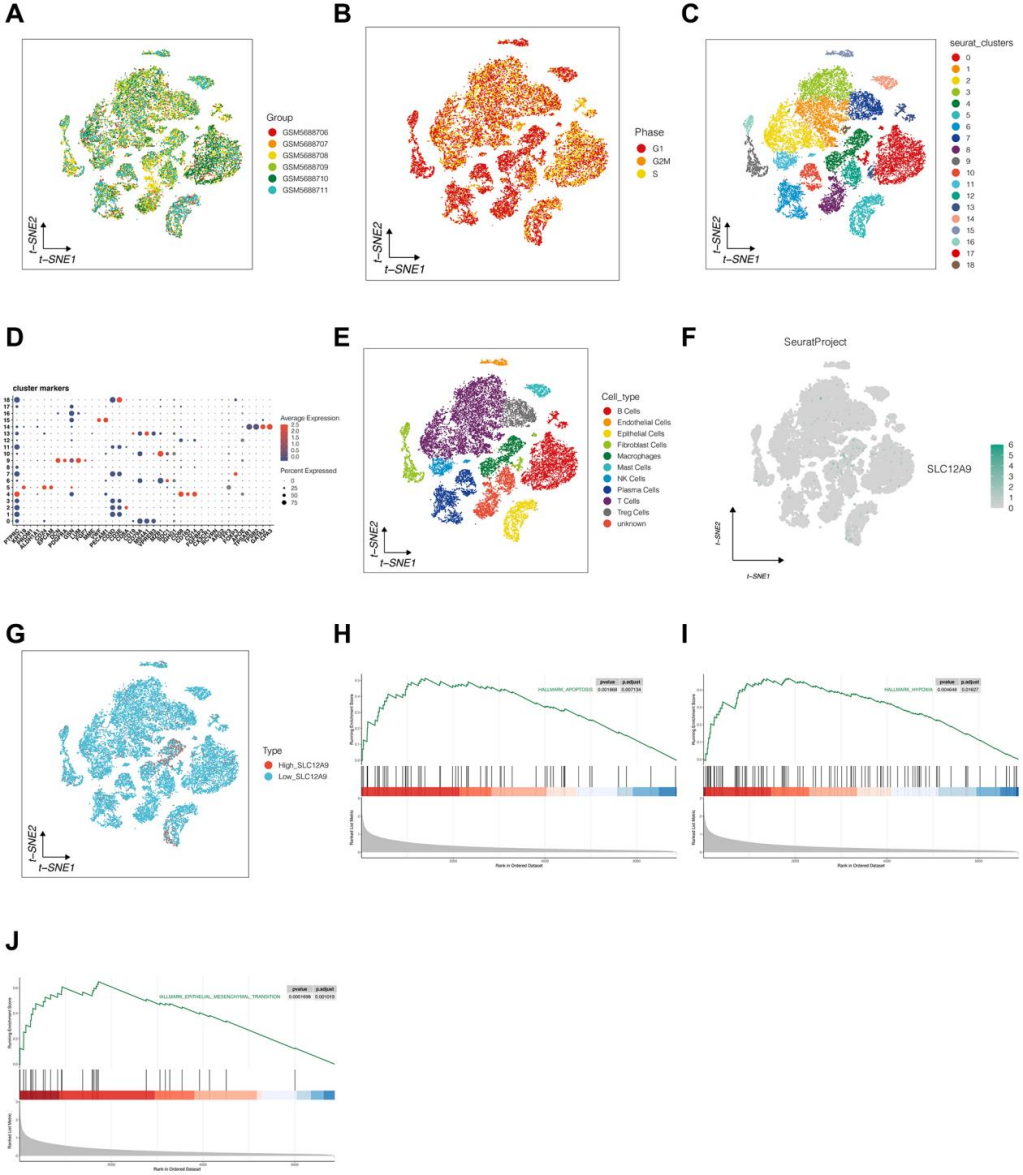
**Single cell analysis and enrichment analysis.** (**A**, **B**) Detection of batch effect. (**C**) The clustering of cells. (**D**, **E**) The annotation of all cells based on the expression of marker genes in each cluster. (**F**, **G**) The expression of *SLC12A9* in different cells. (**H**–**J**) GSEA analysis.

Colorectal cancer single cell datasets were subjected to analysis. In Figure 5A, 5B, a total of 6 samples were utilized in the analysis. No noticeable batch effect was observed within these 6 samples, and the distribution of cell cycle types displayed a relatively even pattern in the TSNE diagram, indicating the suitability of the data for subsequent analysis. Figure 5C demonstrates the clustering of all cells into 19 distinct clusters, with clear gaps between them. Figure 5D, 5E shows the annotation of all cells into 10 cell types based on the expression of marker genes in each cluster. Notably, cluster 8 and cluster 12 exhibited no discernible expression of marker genes, and were therefore categorized as “unknown.” In Figure 5F, 5G, the expression of *SLC12A9* in cells was examined, and cells were classified into high and low expression groups based on the median value. It was observed that cells with high *SLC12A9* expression were primarily macrophages and epithelial cells. To delve deeper into the activation pathway of *SLC12A9*, the FindMarkers function of Seurat was employed to identify marker genes specifically expressed in high-level *SLC12A9* cells, as depicted in Figure 5H–5J. This analysis revealed the activation of apoptosis, hypoxia, and epithelial mesenchymal transition signaling pathways.

### Correlation analysis of immune cell infiltration

**Figure 6:**
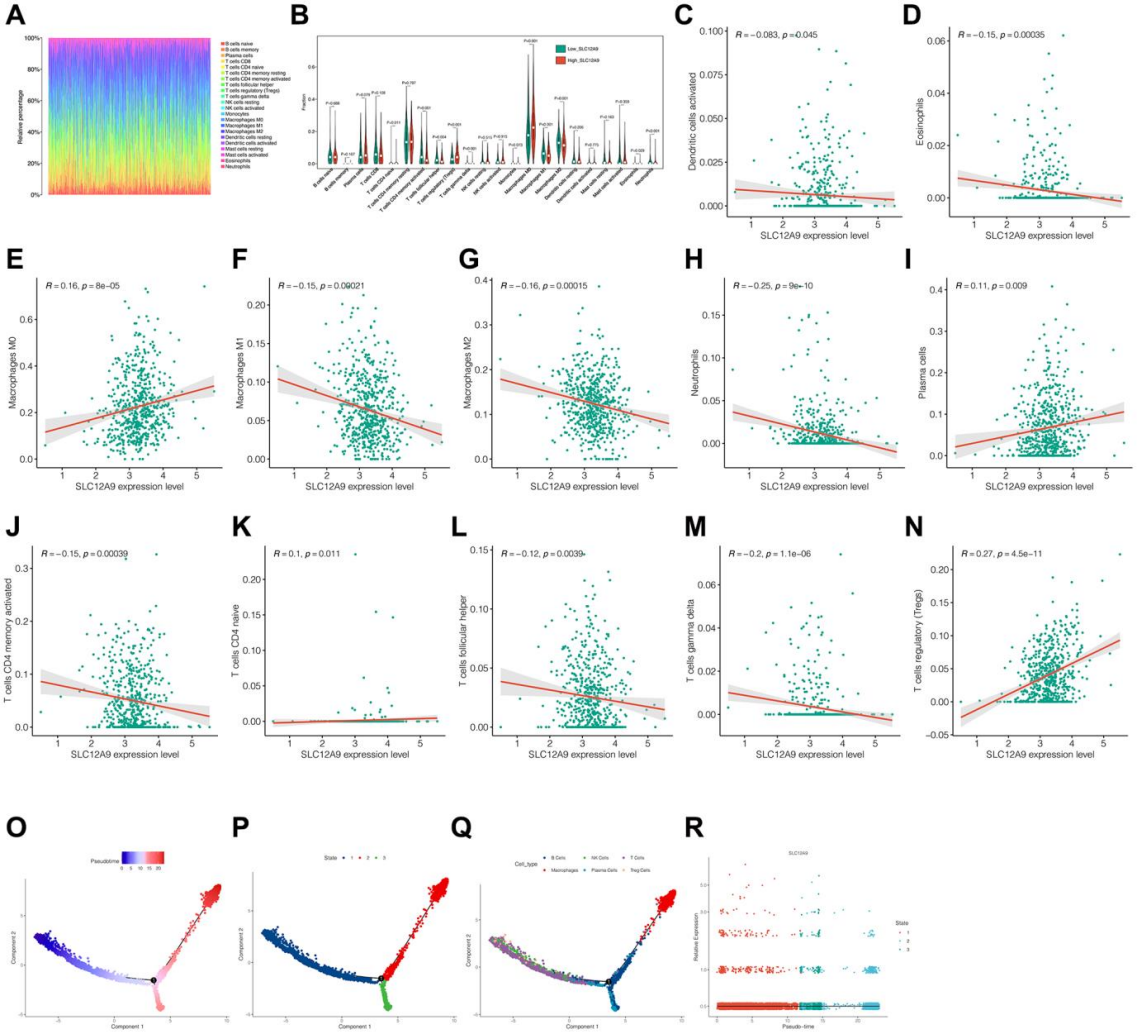
**Correlation analysis of immune cell infiltration.** (**A**) Immune landscape of colorectal cancer sample from the TCGA dataset. (**B**) Infiltration of different immune cells between high and low *SLC12A9*. (**C**–**N**) Correlation analysis between *SLC12A9* and immune cells. (**O**–**R**) Pseudo-time series analysis of immune cells and *SLC12A9*.

The investigation of the immune microenvironment in colorectal cancer was conducted. In Figure 6A, the infiltration of immune cells in each colorectal cancer sample from the TCGA dataset is displayed. Figure 6B reveals the significant upregulation of Macrophages M0, T cells regulatory (Tregs), and Macrophages M0 in the *SLC12A9* group. Conversely, immune cells such as T cells CD4 memory activated, T cells CD4 naive, T cells follicular helper, T cells gamma delta, Neutrophils, and Eosinophils were significantly downregulated (*p* < 0.05). Figure 6C–6N demonstrate the positive correlation between immune cells and the expression of Macrophages M0, plasma cells, T cells CD4 naive, and T cells regulatory (Tregs). Moreover, the expression of *SLC12A9* exhibited a positive correlation with the following immune cells: Dendritic cells activated, Eosinophils, Macrophage M1, Macrophage M2, Neutrophils, T cells CD4 memory activated, T cells follicular helper, and T cells gamma delta (*p* < 0.05). Subsequently, all immune cells were extracted, and time series analysis was simulated, as depicted in Figure 6O–6R. The color blue represents early differentiation of immune cells, while red represents late differentiation. Notably, macrophages were classified into three distinct differentiation states: Macrophage T cells, NK cells, Tregs, and macrophage B cells. State 3 corresponds to plasma cells, and the expression of *SLC12A9* showed no significant changes across different states of immune cell differentiation.

### The construction of Nomogram

**Figure 7:**
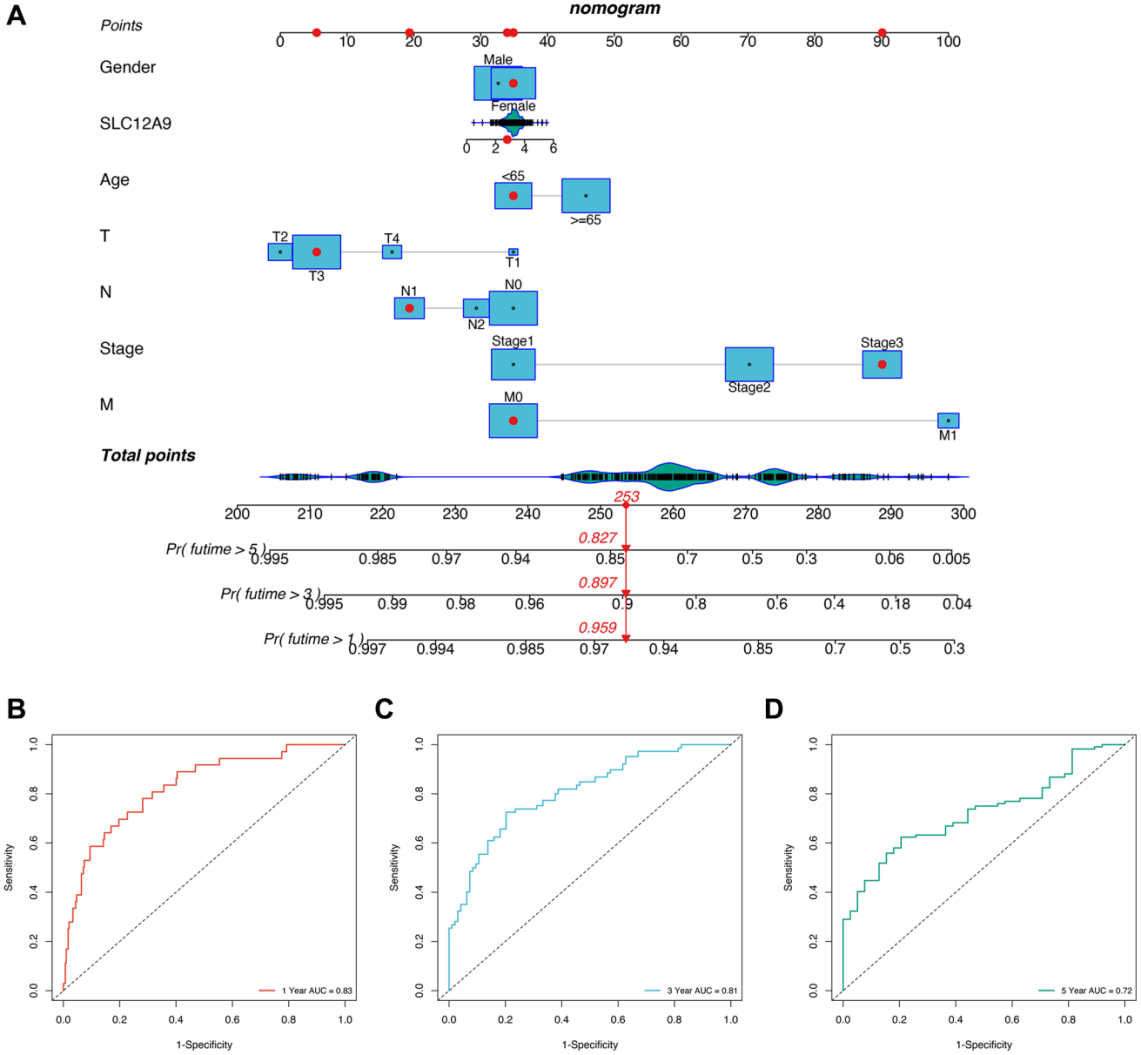
**The construction of Nomogram.** (**A**) The nomogram. (**B**–**D**) Prognostic ROC analysis in 1, 3, and 5-years.

To enhance the prediction of patient prognosis, a Nomogram was constructed based on the expression and clinical characteristics of patients with *SLC12A9*. Figure 7A presents the survival rates of patients with TCGA-G4-6309 at 1, 3, and 5 years, which were 0.959, 0.897, and 0.827, respectively. To further assess the prognostic accuracy of the Nomogram, prognostic ROC analysis was conducted, as depicted in Figure 7B–7D. The AUCs (Area Under the Curve) for the 1, 3, and 5-year prognostic ROC curves were 0.83, 0.81, and 0.72, respectively. These results indicate that the Nomogram exhibited a favorable predictive effect.

### The expression of SLC12A9 in clinical tissue samples was verified by qRT-PCR

**Figure 8:**
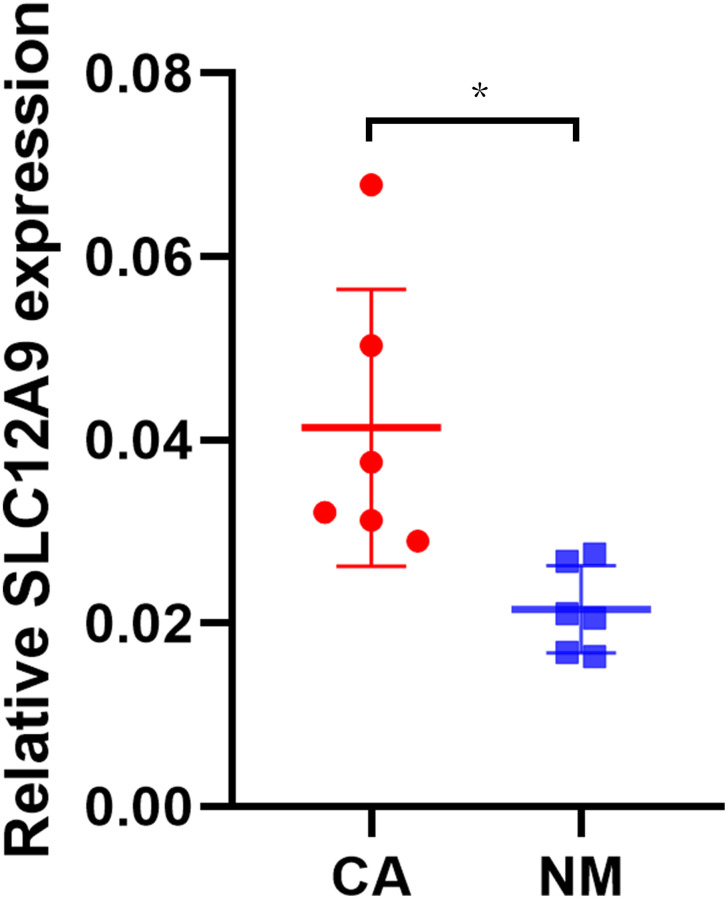
**The qRT-PCR of *SLC12A9*.** The results demonstrated a significant upregulation of *SLC12A9* expression in colon cancer compared to the paired normal tissue samples (*p* < 0.05).

To further validate the findings, qRT-PCR experiments were performed on 6 pairs of clinical colorectal cancer samples and their corresponding normal tissue samples, as illustrated in Figure 8. The results demonstrated a significant upregulation of *SLC12A9* expression in colon cancer compared to the paired normal tissue samples (^*^p < 0.05).

## DISCUSSION

CRC, a major worldwide health concern, contributes significantly to cancer-related mortality [25]. It originates from the epithelial cells of the colon or rectum and is influenced by a combination of genetic and environmental factors [26]. Timely detection of CRC is pivotal for effective treatment and enhanced patient survival rates [27]. However, existing diagnostic techniques like colonoscopy and fecal occult blood tests have drawbacks including invasiveness, high expenses, and limited sensitivity [28].

In addition, the prognosis of CRC exhibits significant variation based on the disease stage during diagnosis. Patients diagnosed with early-stage CRC generally experience more favorable prognoses, whereas those with advanced stages or metastasis encounter significantly poorer outcomes [29]. Hence, the identification of dependable biomarkers that can assist in early detection, risk assessment, and prognostic evaluation holds great significance in the management of CRC.

At present, some studies have preliminarily shown that SLC12A9 plays a key role in colorectal cancer. Yan et al. found that SLC12A9 promotes aggressiveness in uveal melanoma and may be associated with a poor prognosis [30]. However, no bioinformatics studies have explored the role of SLC12A9 in colorectal cancer in depth. This study aimed to comprehensively analyze the clinical significance of *SLC12A9* in CRC using a combination of transcriptome analysis, single-cell sequencing, and qRT-PCR validation. Through the examination of *SLC12A9* expression patterns and its potential diagnostic and prognostic relevance, valuable insights were obtained regarding its potential as a biomarker for CRC.

The findings of this study provide important information on the clinical utility of *SLC12A9* as a potential biomarker for CRC diagnosis and prognosis. The analysis of *SLC12A9* expression, supported by transcriptome analysis, single-cell sequencing, and qRT-PCR validation, highlights its potential as a diagnostic indicator for CRC. The observed upregulation of *SLC12A9* in CRC tissues compared to adjacent normal tissues suggests its involvement in the development of CRC. Detecting elevated levels of *SLC12A9* may enable clinicians to identify individuals at high risk of CRC, facilitating early intervention and ultimately leading to improved patient outcomes.

Additionally, our study uncovered the prognostic value of *SLC12A9* in CRC, which is a significant finding. Patients with elevated *SLC12A9* expression demonstrated inferior overall survival and disease-free survival, highlighting its potential as a prognostic biomarker. The independent prognostic significance of *SLC12A9* further supports its value in risk stratification and guiding personalized treatment decisions. Integrating the assessment of *SLC12A9* expression into existing prognostic models enables clinicians to better evaluate the aggressiveness of the disease and make informed decisions regarding tailored treatment approaches.

The clinical significance of our study lies in the potential practical application of *SLC12A9* in routine clinical practice. The utilization of *SLC12A9* as a target in non-invasive diagnostic tests, such as blood-based assays or stool-based tests, could revolutionize CRC screening and monitoring. These tests would offer enhanced sensitivity and specificity, enabling the early detection of CRC and its precancerous lesions. Moreover, incorporating *SLC12A9* expression into existing prognostic models could augment their accuracy and provide more precise risk stratification for CRC patients, facilitating personalized treatment approaches and post-treatment care.

In conclusion, our study contributes to the growing body of evidence highlighting the clinical value of *SLC12A9* as a diagnostic and prognostic biomarker in CRC. The dysregulated expression of *SLC12A9* in CRC tissues, along with its association with clinicopathological features and patient outcomes, underscores its potential as a valuable tool in CRC management. Integrating *SLC12A9* into clinical practice has the potential to enhance CRC screening, risk assessment, and treatment decision-making, ultimately leading to improved patient outcomes and a reduction in the global burden of CRC. However, there are some limitations to our study. We only have PCR validation of the samples. There is a lack of corresponding cohort for prognostic verification. At the same time, we did not study the downstream mechanism. We will improve it in the future.
